# Obesity epidemic has emerged among Nigerians

**DOI:** 10.1186/1471-2458-14-455

**Published:** 2014-05-15

**Authors:** Sally N Akarolo-Anthony, Walter C Willett, Donna Spiegelman, Clement A Adebamowo

**Affiliations:** 1 Department of Nutrition, Harvard School of Public Health, Boston, MA 02115, USA; 2 Office of Strategic Information and Research, Institute of Human Virology, Abuja, Nigeria; 3 Department of Epidemiology, Harvard School of Public Health, Boston, MA 02115, USA; 4 Department of Biostatistics, Harvard School of Public Health, Boston, MA 02115, USA; 5 Institute of Human Virology and Greenebaum Cancer Center, University of Maryland School of Medicine, Baltimore, MD 21201, USA

**Keywords:** Overweight, Obesity, Nigeria

## Abstract

**Background:**

Data from the WHO shows that the prevalence of overweight and obesity increased by ~20% between 2002 and 2010 in Nigeria. We conducted this study to examine the correlates of this fast growing epidemic.

**Methods:**

We conducted a cross-sectional study among a random sample of 1058 adults, who were visitors and staff of a government worksite in Abuja, an urban city in Nigeria. The study participants had varying socio-economic status and a wide range of occupations, including skilled labor and professionals. Log-binomial regression models were used to estimate the multivariable-adjusted associations of potential determinants with the prevalence of overweight and obesity.

**Result:**

The mean age and body-mass index of the study population were 42 years ± (9.3) and 27 kg/m^2^ ± (4.8). The overall prevalence of overweight or obesity (body-mass index ≥ 25 kg/m^2^) was 64% (74% of the women and 57% of the men). For women compared to men, the prevalence ratio (PR) and (95% confidence interval, CI) was 1.24 (95% CI 1.08, 1.43, p = 0.004), for overweight, and 2.54 (95% CI 2.08, 3.10, p = <0.0001), for obesity. Individuals aged 40 – 49 years were more likely to be overweight or obese. The PR for overweight and obesity was 1.45 (95% CI 1.07, 1.97), p for age trend = 0.002 and 8.07(95% CI 3.01, 21.66, p for age trend = <0.0001) for those aged 40 – 49 years, compared with those aged <30 years. Compared with the individuals in the lower socio-economic status, the PR for obesity among those in the middle and high socio-economic statuses, were 1.39 (95% CI 1.13, 1.72) and 1.24 (95% CI 0.97, 1.59) respectively, p for trend = 0.003.

**Conclusion:**

About two-thirds of urban, professional, high socio-economic status Nigerian adults are either overweight or obese. The prevalence of overweight and obesity among this population of adult Nigerians, is as high as it is in the United Kingdom. Female gender and older age were independent predictors of overweight and obesity; while middle or high socio-economic status were independently associated with obesity.

## Background

Overweight and obesity, defined by the WHO as body-mass index ≥ 25 kg/m^2^ and ≥30 kg/m^2^, respectively, are associated with several diseases including cardiovascular disease, diabetes and several types of cancer cancer and cardiovascular disease [1]. In 2008, 1.4 billion adults aged 20 years and above were overweight or obese, of whom approximately 200 million men and 300 million women were estimated to be obese [2]. Although it had been projected that by 2030, there will be 2.16 billion overweight and 1.12 billion obese individuals globally [3], results from trend analysis suggest that 2 or more billion people worldwide are currently overweight or obese [4]. High blood pressure and high fasting plasma glucose were leading risk factors for disease worldwide in 2010 [1] and are in part caused by obesity. The global disease burden attributable to high body-mass index increased from 52 million in 1990 to 94 million disability-adjusted life-years in 2010 [1].

Although more people in developing countries now die from obesity-associated diseases, including ischemic heart disease, diabetes mellitus and cancer [5], many people are still under the impression that overweight and obesity affects only the Western world and that lower resource countries continue to struggle with only underweight, malnutrition and infections. This may not be the case because the obesity epidemic is growing faster in developing countries than in the developed world [6]. Among people aged 15 years and above, the WHO estimated that the prevalence of overweight and obesity in 2010 was as high as 63.8% and 21.3% respectively, for men, and 73.8% and 43.2% respectively, for women, in some Sub-Saharan Africa countries. Eritrea, Ethiopia, Democratic Republic of the Congo and Central African Republic had the lowest prevalence, while Seychelles, Lesotho, South Africa and Mauritius had the highest prevalence of overweight and obesity in Sub-Saharan Africa [7]. In general, the countries with lower prevalence of overweight and obesity tend to be those with low gross domestic product per capita and vice versa, suggesting that socio-economic status may be a determinant of overweight and obesity in some African countries.

According to the 2010 WHO survey data on Nigeria, the prevalence of overweight was 26% and 37% in men and women respectively, while the prevalence of obesity was 3% and 8.1% in men and women respectively [7]. Data from the WHO Global InfoBase, based on individuals aged 30 years and above, shows that the prevalence of overweight and obesity together increased by 23% in men and 18% in women, while the prevalence of obesity alone increased by 47% in men and 39% in women, between 2002 and 2010, in Nigeria [7].

Previous studies have shown that several factors including age, gender, marital and socio-economic statuses, occupation, urban residence, dietary intake and physical activity are associated with overweight and obesity [8-12]. Even though the prevalence of overweight and obesity in Nigeria continues to increase, there are few studies of its correlates. We therefore conducted this study to examine the prevalence of overweight and obesity and its potential correlates in an urban Nigerian population.

## Methods

### Study population

Between April 2010 and February 2011, we conducted a cross-sectional study among a random sample of 1058 workers and visitors at the Federal Secretariat Complex, a government worksite in Abuja, Nigeria, which houses the offices of federal public sector workers in central Nigeria. Because it is a federal establishment, the staff distribution is representative of Nigeria’s ethnic and cultural diversity. We approached individuals aged over 18 years who reside within Abuja city to participate in the study; they had a wide range of occupations including skilled labor and professionals.

### Demographic, socio-economic factors and physical activity

To verify that we had sampled a diverse population, we collected data on ethnicity, religion, level of education, and profession. To evaluate socio-economic status, we asked about household possessions including fan, refrigerator, television, bicycle, motorcycle, car, source of drinking water, type of sanitation, type of residence, home ownership, separate room for cooking, source of cooking fuel, respondent self-reported social class and interviewer-perceived social class. To assess physical activity levels, we asked about amount of time spent on different exercises/week, as defined in the WHO global physical activity recommendations for individuals aged 18 – 64 years [13].

### Anthropometric measurements

Trained research nurses measured individual height with a rigid tape measure, in accordance to the World Health Organization (WHO) multinational monitoring of trends and determinants in cardiovascular disease criteria [14]. To measure height, the participants’ were asked to take off his/her shoes, hats or head ties; stand with back to the tape measure; and hold their head in a position where he/she can look straight at a spot, head high, on the opposite wall. A flat rule was placed on the participant’s head, so that the hair (if present) was pressed flat. Height was measured to the nearest centimeter, at the level where the flat rule touched the rigid tape.

To measure weight, participants’ were asked to remove heavy outer garments, empty their pockets and step on a weighing scale, which was placed on a hard, even surface. Weight was estimated using the Omron body sensor (Omron HBF-510 W Full Body Sensor Body Composition Monitor Scale). Body-mass index was estimated as a ratio of an individual’s weight (kg)/height (m^2^). Body-mass index categories were defined using the WHO cut points in units of kg/m^2^, normal weight = 18.5 - < 25, overweight = 25 - < 30 and obese ≥ 30.

### Dietary measurements

The method of estimating calorie intake has been described elsewhere [15]. Briefly, dietary carbohydrate intake over the previous 1 year was estimated, with a modified semi-quantitative food frequency questionnaire. The food frequency questionnaire collected information on 11 main foods and 7 beverages. Participants were asked to report types of foods and beverages consumed; the frequency of consumption (number of times consumed daily, weekly and monthly); and the quantity consumed. We used the Nigerian food composition database [16] to translate dietary intake to calorie intake.

### Statistical analysis

A total of 1058 participants were enrolled in this study. We excluded 17 persons who were underweight, defined as having a body-mass index < 18.5 kg/m^2^. We generated a wealth index using the factor analysis (principal components) procedure and varimax rotation as previously described by Filmer and Pritchett [17], to compute socio-economic status.

We used mean and standard deviation (SD) for continuous variables while *t*-tests were used to assess the significance of differences between groups in the distribution of continuous variables; *χ*^2^ tests were used for categorical variables; Spearman and Pearson correlation coefficients were used to assess the correlation between the covariates.

Univariate and multivariate analyses with log-binomial regression models were conducted to examine the associations between potential correlates and the prevalence of overweight and obesity [18-20]. All variables that were associated with overweight and obesity in the univariate analyses with p-value ≤ 0.2, were included in the multivariate model [21]. Stepwise restricted cubic splines were used to assess and graph non-linearity [22]. Tests for non-linearity used the likelihood ratio test, comparing the model with only the linear term to the model with the linear and the cubic spline terms [23]. The fit of the models were assessed with Akaike information criterion. All analyses were conducted with SAS for UNIX statistical software (version 9.2; SAS Institute).

### Ethics

The study was conducted according to the Nigerian National Code for Health Research Ethics and the Declaration of Helsinki. Ethical approval to conduct this study was obtained from the Institute of Human Virology Nigeria Health Research Ethics Committee. Individuals were informed about the study and were requested to consent before participating in the study.

## Result

The mean age (SD) of the participants was 41.6 (9.3) years; 40% (416/1041) were women and 60% (625/1041) were men. The mean (SD) body-mass index of all the participants was 27.2 (4.8) kg/m^2^; 36% (377/1041) of the study participants were of normal weight 38% (399/1041) were overweight and 26% (265/1041) were obese. The mean (SD) body-mass index was 22.5 (1.6) kg/m^2^ for the participants within the normal weight range, 27.2 (1.4) kg/m^2^ among the overweight and 33.8 (3.2) kg/m^2^ for those who were obese. Table 1 shows the demographic characteristics of the study population overall and by body-mass index categories. Some 32% (134/416) of the women and 42% (265/625) of the men were overweight, while 42% (174/416) of the women and 15% (94/625) of the men were obese. Table 2 shows the prevalence of overweight and obesity by gender.

**Table 1:** Characteristics of the study population by body-mass index categories

	**Overall %**	**Body-mass index categories (kg/m**^**2**^**)**		
**Characteristics**		18.5 < 25	25 - < 30	≥ 30.0
		n = 377	n = 399	n = 265
**Body-mass index (kg/m**^**2**^**)**		22.5 ± 1.6	27.2 ± 1.4	33.8 ± 3.2
**Age (years)**		39.2 ± 10.3	42.6 ± 9.0	43.3 ± 7.1
**Age categories (years), %**				
- <30	11	19	10	2
- 30 – 39	28	31	22	32
- 40 – 49	40	32	46	43
- ≥ 50	21	18	22	23
**Sex,%**				
- Male	60	71	67	34
- Female	40	29	33	66
**Religion, %**				
- Christianity	80	78	80	82
- Islam	20	22	20	18
**Marital Status, %**				
- Married	77	69	81	82
- Not married	23	31	19	18
**Education, %**				
- None	0.4	0	1	0
- Primary (Elementary school)	0.6	1	1	0
- Secondary (High school)	22	29	19	15
- ≥ Tertiary (College)	77	70	79	85
**Occupation, %**				
- Self-employed	3	4	2	1
- Unskilled manual	7	11	6	3
- Skilled manual	40	43	40	36
- Professional/executive	50	42	52	60
**Socio-economic status, %**				
- Low	40	51	38	27
- Middle	40	31	41	52
- High	20	18	21	21
**Sugar sweetened beverages, %**				
- < 1/month	17	17	15	20
- ≤ 1/week	45	44	48	40
- 2 – 6/week	27	27	27	27
- ≥ 1/day	11	12	10	13
**Mean calories from carbohydrates**		985.7 ± 502.7	973.4 ± 497.8	862.8 ± 482.4
**Physical activity, %**				
- Low	61	59	61	65
- Moderate	22	23	23	20
- High	16	18	16	15
**Television (hours/week), %**				
- <6/week	45	46	44	46
- 6 - 10/week	28	29	28	26
- 11 - 20/week	17	15	19	18
- > 20/week	10	10	9	10

**Table 2:** Prevalence of overweight and obesity among urbanized adult Nigerians, by gender

**Body-mass index category**	**Body-mass index cut-off point**	**Women (%)**	**Men (%)**
Normal weight	18.50 – 24.99	26	43
Overweight	25.00 – 29.99	32	42
Obese	≥ 30.00	42	15

**Figure 1:**
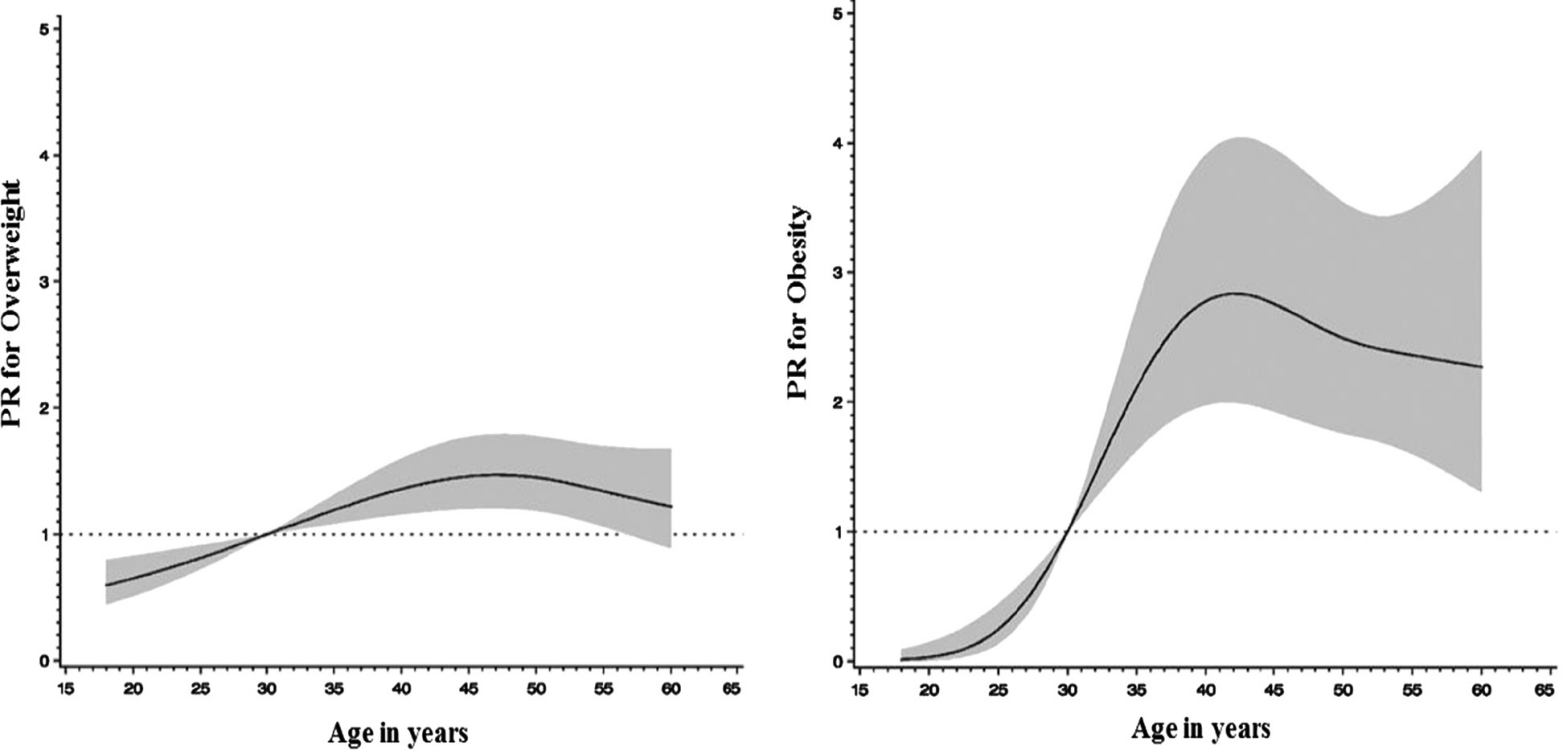
The prevalence of obesity and overweight in relation to age.

Individuals who were older than 30 years were more likely to be overweight or obese than younger persons. Compared to individuals aged < 30 years, the prevalence ratio (PR) for being overweight was 1.45 (95% CI = 1.07, 1.97) for individuals aged 40 – 49 years and 1.35 (95% CI = 0.98, 1.85, p-value for trend test = 0.002) for individuals aged 50 years and above; while the PR for obesity was 8.07 (95% CI = 3.01, 21.66) for individuals aged 40 – 49 years and 7.74 (95% CI = 2.88, 20.81 p-value for trend test = <0.0001) in individuals who are 50 years of age and above. The prevalence of overweight and obesity was related to age in a non-linear manner (p < 0.001). Both conditions increased linearly with age up to around age 45, at which point the prevalence of these conditions remained constant or even declined (Figure 1). Comparing women to men, the PR and 95% CI for overweight and obesity were 1.24 (95% CI = 1.08, 1.43 p = 0.004) and 2.54 (95% CI = 2.08, 3.10 p = <0.0001, respectively. Being married was also associated with increased prevalence of overweight and obesity. Table 3 shows the results of univariate and multivariate analyses of the risk factors for the prevalence of overweight while Table 4 shows the same information for obesity.

**Table 3:** Univariate and multivariate analyses of risk factors for overweight vs. normal weight

**Characteristics**	**N**	**Univariate**		**Multivariate**	**p-value**
		**RR (95% CI)**	**p-value**	**RR (95% CI)**	
**Age categories (years)**			<0.0001		0.002
- <30	113	1.00		1.00	
- 30 – 39	206	1.23 (0.92, 1.66)		1.11 (0.81, 1.52)	
- 40 – 49	301	1.71 (1.31, 2.23)		1.45 (1.07, 1.97)	
- ≥ 50	156	1.58 (1.18, 2.10)		1.35 (0.98, 1.85)	
**Sex**			0.17		0.004
- Male	533	1.00		1.00	
- Female	241	1.11 (0.96, 1.27)		1.24 (1.08, 1.43)	
**Marital status**			<.0001		0.043
- Not married	193	1.00		1.00	
- Married	583	1.43 (1.18, 1.73)		1.24 (0.99, 1.54)	
**Education**			0.02		0.46
- Secondary (High school)	186	1.00			
- ≥ Tertiary (College)	582	1.32 (1.09, 1.59)		1.13 (0.92, 1.39)	
- None/Primary (Elementary)	8	1.59 (0.61, 3.47)		1.39 (0.30, 3.82)	
**Occupation**			0.007		0.68
- Skilled manual	323	1.00			
- Professional/executive	364	1.14 (0.99, 1.32)		1.00 (0.86, 1.16)	
- Unskilled manual	65	0.71 (0.51, 1.01)		0.84 (0.59, 1.18)	
- Self-employed	24	0.84 (0.52, 1.37)		0.79 (0.38, 1.64)	
**Socio-economic status**			0.0007		0.10
- Low	342	1.00		1.00	
- Middle	280	1.34 (1.14, 1.56)		1.18 (1.01, 1.39)	
- High	154	1.26 (1.04, 1.52)		1.15 (0.95, 1.39)	
**Sugar sweetened beverages**			0.60		
- < 1/month	126	1.00			
- ≤ 1/week	356	1.10 (0.90, 1.35)			
- 2 – 6/week	210	1.07 (0.86, 1.34)			
- ≥ 1/day	84	0.96 (0.72, 1.28)			
**Physical activity**			0.61		
- Low	466	1.00			
- Moderate	180	0.97 (0.82, 1.15)			
- High	130	1.91 (0.74, 1.11)			
**Television (hours/week)**			0.49		
- <6/week	350	1.00			
- 6 - 10/week	219	1.00 (0.85, 1.18)			
- 11 - 20/week	133	1.13 (0.94, 1.35)			
- > 20/week	74	0.94 (0.72, 1.22)			

**Table 4:** Univariate and multivariate analysis of risk factors for obesity vs. normal weight

**Characteristics**	**N**	**Univariate**	**p-value**	**Multivariate**	**p-value**
		**RR (95% CI)**		**RR (95% CI)**	
**Age categories (years)**			<0.0001		<0.0001
- <30	77	1.00		1.00	
- 30 – 39	201	8.14 (3.09, 21.43)		6.73 (2.51, 18.02)	
- 40 – 49	234	9.46 (3.61, 24.78)		8.07 (3.01, 21.66)	
- ≥ 50	130	9.03 (3.42, 23.87)		7.74 (2.88, 20.81)	
**Sex**			<.0001		<0.0001
- Male	358	1.00		1.00	
- Female	282	2.43 (1.99, 2.96)		2.54 (2.08, 3.10)	
**Marital status**			0.0001		0.009
- Not married	166	1.00		1.00	
- Married	476	1.58 (1.22, 2.04)		1.24 (1.00, 1.53)	
**Education**			0.0003		0.83
- Secondary (High school)	148	1.00		1.00	
- ≥ Tertiary (College)	489	1.74 (1.31, 2.31)		1.03 (0.80, 1.34)	
- None/Primary (Elementary)	4	1.80 (0.26, 7.33)		1.21 (0.38, 5.42)	
**Occupation**			<.0001		0.008
- Skilled manual	259	1.00		1.00	
- Professional/executive	317	1.35 (1.12, 1.64)		1.25 (1.06, 1.48)	
- Unskilled manual	50	0.43 (0.22, 0.83)		0.69 (0.36, 1.31)	
- Self-employed	16	0.34 (0.09, 1.24)		0.47 (0.13, 1.65)	
**Socio-economic status**			<0.0001		0.003
- Low	263	1.00		1.00	
- Middle	254	2.01 (1.60, 2.53)		1.39 (1.13, 1.72)	
- High	125	1.66 (1.26, 2.19)		1.24 (0.97, 1.59)	
**Sugar sweetened beverages**			0.93		
- < 1/month	119	1.00			
- ≤ 1/week	272	0.86 (0.67, 1.10)			
- 2 – 6/week	173	0.92 (0.70, 1.19)			
- ≥ 1/day	78	0.93 (0.67, 1.29)			
**Physical activity**			0.26		
- Low	393	1.00			
- Moderate	142	0.87 (0.68, 1.10)			
- High	107	0.83 (0.63, 1.10)			
**Television (hours/week)**			0.65		
- <6/week	294	1.00			
- 6 - 10/week	177	0.95 (0.75, 1.19)			
- 11 - 20/week	106	1.12 (0.88, 1.44)			
- > 20/week	65	0.97 (0.70, 1.35)			

Higher occupational level and a higher socio-economic status were significantly associated with an increased prevalence of obesity but not overweight. The PR for obesity among those in the middle and high socio-economic categories, were 1.39 (95% CI 1.13, 1.72) and 1.24 (95% CI 0.97, 1.59) respectively, p for trend = 0.003, compared with the lower socio-economic status category

## Discussion

Only 36% of the participants in this study had normal body-mass index, the remaining 64% were either overweight or obese. Factors that were significantly associated with higher prevalence of overweight and obesity were being in older age groups, and female gender. In addition, having a more professional job and belonging to middle or high socio-economic status were significantly associated with increased obesity prevalence.

Some 77% of our study participants had at least a college degree and 50% had professional jobs. Data from the 2008 Nigeria Demographic and Health Survey [24] shows that Abuja, federal capital territory, has the highest proportion of men and women who have completed a secondary school education, among all 36 states and the federal capital territory, in Nigeria. Our findings are consistent with studies on obesity in Sub Saharan Africa, that the prevalence of obesity was more marked among urban, highly educated women [8], who are more likely to have sedentary lifestyles and access to processed foods, compared to women in rural areas.

The findings from our spline analysis showed that, the prevalence ratio of obesity increases rapidly after age 30 years and peaks at age 40 – 49 years. Our findings, on the prevalence of overweight and obesity among adults aged 30 years or older in Nigeria, were higher than in the WHO 2010 survey [25]. However, unlike our study which was based on an urban population, the WHO survey included rural and urban populations. Our estimates were also substantially higher than estimates from other recent studies in Nigeria. Table 5 shows how the prevalence estimates obtained from these studies, compare to our prevalence estimates and the setting for each study population. In contrast to these studies, our study sample was urban, more educated and more likely to be engaged in professional vocations compared to average Nigerians [24]. As the Nigerian economy continues to improve, food availability and individuals’ dietary calorie intake may have increased, thus contributing to more people becoming overweight and obese. People living in rural Nigeria still consume more traditional Nigerian diets which have more whole grains, have higher fiber and fewer calories. They may also have less sedentary lifestyles compared to those in the urban areas. However, there is scarce data, on the nutritional and physical activity patterns, among Nigerians.

**Table 5:** Summary of recent prevalence studies on overweight and obesity among adult Nigerians

**Ref.**	**Year(s) of data collection**	**Location in Nigeria**	**Sample size**	**Age range**	**Mean age (SD)**	**Overweight (%)**	**Obesity (%)**
Present Study	2010 - 2011	Abuja, Central, (Urban)	1041	18 - 65	41.6 (9.3)	38	26
[26]	2010 - 2011	Maiduguri, North-east, (Urban)	1818	20 - 65	32.3 (10)	22.8	8.1
[27]	-	Abia, South-east, (Semi-rural)	1584	18 - 29	21.8 (2.1)	20.7	-
[28]		Lagos, South-west, (Urban)	900	20 - 80	37.8 (14.3)	29	9
[29]	2008 - 2009	Imo, South-east, (Rural)	2156	18 - 90	42.6 (11.3)	-	6
[30]	-	Benue, (Rural & Urban)	435	18 - 45	24.2 (0.2)	22.1	3.9
[31]	2006	Katsina, North-west, (Urban)	300	-	37.6 (10.6)	53.21	21
[9]	-	Ibadan, South-west, (Urban)	998	19 - 70	40.0 (8.3)	17.4	8.7

Our results are consistent with other African studies, that overweight and obesity is more prevalent among women, persons older than 30 years and married individuals [9,11,12,28,32]. The gender disparity among overweight and obesity is not well understood. However, weight gain during and after pregnancy, the perception of weight gain as evidence of high socio-economic status [33], fattening practices among women [34,35] and increased prevalence of more sedentary occupations among Sub-Saharan African women, may contribute to this. Although women in Africa may do more household chores, men’s professions and work related to engaging in these occupations, participation in sports and other physical activities, may contribute to the different prevalence between men and women [15]. In addition, Nigerian women in urban areas who are highly educated and have higher socio-economic status, may not engage in household chores as they are more likely to have maids who do the work.

Higher socio-economic status and occupational level were significantly associated with the risk of obesity. This finding was consistent with the pattern of results in low- and middle-income countries [10]. We noted that those that were at least moderately physically active were more likely to be overweight or obese, even though this was not statistically significant. Some of these people may include those trying to lose weight. Also, obesity and overweight was more prevalent among those reporting consumption of fewer carbohydrate calories. This may be due to underreporting of dietary intake by those who were overweight and obese [36,37]. People with higher dietary calorie intake are also likely to be more physically active. Unlike studies done in developed countries where hours spent watching TV is associated with obesity [38], we did not find this to be the case in this study population.

The prevalence of overweight and obesity in Abuja is high, compared to other cities in Nigeria [9,26-30] but it is similar to the prevalence in developed countries like the United Kingdom, where a greater proportion of men than women (42% compared with 32%) were classified as overweight and 26% of the adults were classified as obese in 2010 [39]. This dramatically increasing prevalence is of particular concern, in view of increased risk of non-communicable diseases such as hypertension, diabetes and cancer. Our findings argue for public health interventions that seek to promote healthy diets and increase physical activity, among the rapidly urbanizing populations, particularly of the developing world.

Our study is limited by its cross-sectional design and its setting in an adult urban population, thus the results cannot be generalized to rural populations or children. As there were many different ethnic groups, we did not have sufficient power to address the role of these unique ethnicities on overweight or obesity. Also, dietary carbohydrate intake was assessed over the past year, which may not be the best representation of the participants’ long-term intake. However, we made objective measurements of anthropometry, using standardized guidelines and techniques, and we found correlates of overweight and obesity in this Nigerian population, that were similar to other highly urban populations in West Africa [9,11,12].

## Conclusion

In conclusion, our findings emphasize the importance of implementing population-wide approaches to reduce diseases associated with increasing urbanization such as obesity in rapidly developing countries like China and Nigeria. Future studies on diet, physical activity and their determinants in urban populations are urgently needed. Specific urban public health policies to promote awareness especially among young adults, education, nutritional interventions, improved urban neighborhood planning and increased physical activity, should be integrated in national health policies to help control this epidemic in Africa.

## Competing interests

The authors declare that they have no competing interests.

## Authors’ contributions

SA analyzed the data and drafted the manuscript. SA and CA conceived and designed the study. DS provided support for the data analysis and interpretation. CA, DS and WW provided critical reviews of the manuscript. All authors were involved in writing the paper and approval the submitted and final versions.

## Pre-publication history

The pre-publication history for this paper can be accessed here:

http://www.biomedcentral.com/1471-2458/14/455/prepub
